# ROS-Induced mtDNA Release: The Emerging Messenger for Communication between Neurons and Innate Immune Cells during Neurodegenerative Disorder Progression

**DOI:** 10.3390/antiox10121917

**Published:** 2021-11-29

**Authors:** Yuanxin Zhao, Buhan Liu, Long Xu, Sihang Yu, Jiaying Fu, Jian Wang, Xiaoyu Yan, Jing Su

**Affiliations:** Key Laboratory of Pathobiology, Department of Pathophysiology, Ministry of Education, College of Basic Medical Sciences, Jilin University, 126 Xinmin Street, Changchun 130021, China; zhaoyx19@mails.jlu.edu.cn (Y.Z.); liubh20@mails.jlu.edu.cn (B.L.); xulong20@mails.jlu.edu.cn (L.X.); yush19@mails.jlu.edu.cn (S.Y.); fujy21@mails.jlu.edu.cn (J.F.); wjian21@mails.jlu.edu.cn (J.W.)

**Keywords:** mtDNA, ROS, neurodegenerative diseases, cGAS/STING, neuroinflammation

## Abstract

One of the most striking hallmarks shared by various neurodegenerative diseases, including Parkinson’s disease, Alzheimer’s disease and amyotrophic lateral sclerosis, is microglia-mediated and astrocyte-mediated neuroinflammation. Although inhibitions of both harmful proteins and aggregation are major treatments for neurodegenerative diseases, whether the phenomenon of non-normal protein or peptide aggregation is causally related to neuronal loss and synaptic damage is still controversial. Currently, excessive production of reactive oxygen species (ROS), which induces mitochondrial dysfunction in neurons that may play a key role in the regulation of immune cells, is proposed as a regulator in neurological disorders. In this review, we propose that mitochondrial DNA (mtDNA) release due to ROS may act on microglia and astrocytes adjacent to neurons to induce inflammation through activation of innate immune responses (such as cGAS/STING). Elucidating the relationship between mtDNA and the formation of a pro-inflammatory microenvironment could contribute to a better understanding of the mechanism of crosstalk between neuronal and peripheral immune cells and lead to the development of novel therapeutic approaches to neurodegenerative diseases.

## 1. Introduction

Neurodegenerative diseases are rare hereditary disorders of the central nervous system that cause a slowly progressive loss of function of specific neuron populations and their connections. In addition to Alzheimer’s and Parkinson’s diseases (AD and PD), these also include amyotrophic lateral sclerosis (ALS). Among these, AD is formed by the deposition of a proteolytic product of the amyloid precursor protein, called amyloid β-protein (Aβ), which leads to mitochondrial disorders [1]. Aβ isolated from human brains is biochemically heterogeneous in terms of the size of the peptide fragments and their posttranslational modifications [2]. Recent studies have shown that non-protofibrillar precursors in amyloid, particularly soluble oligomers, act as the cause of tissue damage [3]. The majority of AD cases present as the typical primarily amnestic form, with early or prominent visual, frontal, motor or other symptoms [4]. PD is a progressive neurodegenerative disorder, which is defined by its classical motor symptoms that include resting tremors, bradykinesia, rigidity and postural instability. These are also accompanied by the loss of dopaminergic neurons and the presence of the hallmark Lewy pathology [5,6,7,8]. ALS is a multifactorial disease, which is characterized by a progressive loss of motor neurons that eventually leads to paralysis and death [9]. It is characterized by the accumulation of hyper-phosphorylated and ubiquitinated TAR DNA-binding protein 43 (TDP-43) deposits in the brain and spinal cord of patients with this motor neuron disease [10].

Numerous studies have shown that reactive oxygen species (ROS) are closely associated with neurodegenerative diseases. ROS significantly promote the degeneration of neurons by regulating the function of biomolecules. ROS target different biological molecules (DNA, RNA, lipids and proteins) and biological processes (nucleic acid oxidation and lipid peroxidation). The brain requires large amounts of oxygen to function properly and can be considered a factory of free radicals, as well as a hotspot for neurodegenerative diseases. ROS forms involved in neurodegeneration include hydrogen peroxide (H_2_O_2_), superoxide anion (O_2_^−^) and highly reactive hydroxyl radicals (HO·) [11].

Mitochondria are responsible for the production of ATP through the electron transport chain and oxidative phosphorylation. They are also involved in the production of molecules to overcome oxidative stress, programmed cell death and respiratory functions of the cell. Mitochondrial enzymes rich in various oxidoreductases and mitochondrial dysfunction are thought to be responsible for the generation of ROS in the cellular environment [12]. Neuronal mitochondria are more vulnerable to oxidative stress because of the high oxygen demand of neurons; their high lipid content; relatively high content of unsaturated fatty acids, which are more sensitive to oxidative activity; and low level of antioxidant enzymes. Recent studies have shown that mitochondrial oxidative stress occurs in the progression of neurodegenerative diseases. We have summarized some of the advances in research on antioxidants in neurodegenerative diseases in Table 1.

Increasing evidence indicates that mitochondrial dysfunction is associated with a variety of neurodegenerative diseases. Neurodegeneration results in the loss of the normal anatomical structure and function of the human nervous system. One of the main functions of mitochondria is to provide energy in the oxidative respiration of cells. When glucose metabolism or electron transfer is perturbed by genetic or exogenous factors, the respiratory chain complex is compromised, which can cause the inhibition of the electrochemical gradient, deficits in energy production and ultimately neurodegeneration [47,48]. Mitochondrial DNA (mtDNA) also plays an important role in mitochondrial dysfunction. The hypothesis proposed by Markesbery centers on the 13 proteins required by the electron transport chain complexes that are encoded by mtDNA. Because mtDNA is adjacent to the site of oxidative metabolism and lacks the protection of histones and repair mechanisms, it is particularly sensitive against oxidative stress [48]. The damaged neurons lead to mitochondrial dysfunctions, causing damage to their mtDNA that promotes the occurrence and development of neurodegenerative diseases. Recent studies have found that leukocytes flood into the injury site and activate the internal microglia of the central nervous system (CNS); these are collectively referred to as neuroinflammation. To cope with various factors, such as oxidative stress, mtDNA may act to induce neuroinflammation [49,50]. In an inflammatory environment, mtDNA released by neurons acts on neighboring immune cells, such as astrocytes and microglia, the latter of which are the main immune defense line of the CNS and play an increasingly important role in brain. Traditionally, microglia are in a resting state (M0 phenotype) under physiological conditions and play an “immune surveillance” role. Under pathological conditions, microglia are rapidly activated, and the activation is accompanied by changes in transcriptional adaptive functions. Neuroinflammatory (M1 polarization) microglia release pro-inflammatory factors and toxic substances to kill pathogens. The neuroprotective (M2 polarization) microglia achieve neuroprotection by promoting tissue repair and regeneration. Clinical studies have shown that overactivated M1 phenotype microglia can cause neuronal disability, damage and degeneration; and play an important role in cerebrovascular and neurodegenerative diseases and neurodevelopmental and mental disorders [51,52,53,54]. This binary M1/M2 classification of macrophage remains widely used, although it represents a simplified view of macrophage phenotype and function, since the neuroinflammatory microenvironment has a rather complex combination of stimuli. Yu et al. confirmed that microglia of the CD11b^+^/CD45 low/high phenotype play an important role in neuroinflammation [55]. Astrocytes are the most numerous cells in the CNS and can help maintain neuronal functions and the stabilization of the CNS, and also can reabsorb neurotransmitters. Under stress, astrocytes proliferate into reactive astrocytes with different phenotypes and functions: Type A1 is biased towards inflammatory activation and can induce neuronal cell death; Type A2 tends to inhibit inflammation, upregulate neurotrophic factors, promote neuron survival and has a protective effect [56].

Neuroinflammation is a key driver of neurodegenerative diseases and can trigger and exacerbate neuronal damage. Here, we describe the role of mtDNA release induced by stress injury in the formation of an inflammatory microenvironment in neurons. We further investigated the mechanism by which mtDNA promotes neuroinflammation, providing a new strategy for the development of targeted drugs, and the treatment of neurodegenerative diseases.

## 2. ROS-Induced Mitochondrial Dysfunction Promotes Neurodegeneration through the Release of mtDNA

### 2.1. Possible Mechanisms of mtDNA Release

“Leaky” mtDNA from damaged mitochondria caused by mitochondrial dysfunction is a significant source of mitochondrial danger-associated molecular patterns (mtDAMPs). Mitochondria share several features with bacteria, including a double membrane structure and a circular genome with non-methylated CpG sites independent of nuclear DNA replication. Given this similarity, once released into the cytoplasmic or extracellular space, mtDNA fragments activate innate immunity and inflammation, such as DAMPs, which are similar to pathogen-associated molecular patterns (PAMPs). This occurs through a molecular cascade reaction that includes binding to Toll-like receptor 9 (TLR9) and subsequent activation of stimulators of the interferon gene (STING) pathway [57]. We speculate that accumulation of DAMPs in neurons may activate microglia and astrocytes and promote leukocyte infiltration.

**Figure 1:**
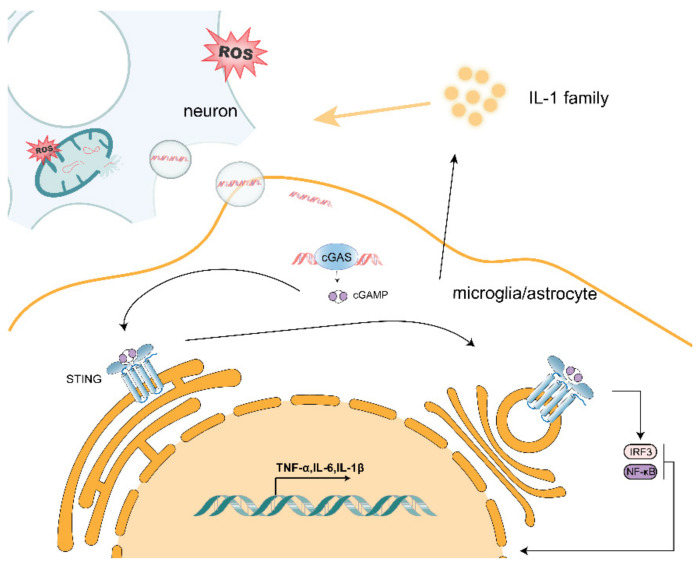
Release of mtDNA from neurons causes activation of the cGAS/STING pathway in neighboring microglia and astrocytes, leading to increased transcription of pro-inflammatory factors.

ROS production is thought to be an upstream step in the oxidative damage of mitochondrial proteins, membranes and mtDNA. This is partly due to the fact that mitochondria are a major source of endogenous ROSs, which are produced in the mitochondrial matrix and escape from metabolic processes and electron transport chains during oxidative phosphorylation. ROSs are produced by electron transport chains and metabolic redox reactions, which can result in mtDNA mutations or deletions, oxidative damage to the respiratory chain, lipid peroxidation and overall mitochondrial dysfunction. When neurons are exposed to oxidative stress, their internal mitochondria produce ROS, leading to mitochondrial dysfunction and the possible release of oxidized mtDNA (Figure 1). Moreover, mtDNA is located in environments with high levels of ROS, and accumulated mtDNA can lead to organelle and cellular dysfunction. Thus, ROS-induced mitochondrial damage may cause the release of mtDNA, which ultimately leads to mitochondrial dysfunction and in turn, chronic inflammation and disease. As demonstrated by Zhao et al. [58], mtDNA damage can be aggravated by mitochondrial ROS. Mitochondrial Lon is a molecular chaperone and DNA-binding protein that plays a role in protein quality control and stress-response pathways. Lon levels regulate mtDNA metabolism and mitochondrial ROS production. Overexpression of Lon induces mitochondrial ROS to oxidize mtDNA, thereby allowing it to be released it into the cytoplasm [59].

As a DAMP, mtDNA can cause cellular stress. Many studies have been conducted on how mtDNA is released into the cytoplasm/extracellular space. Kim et al. found that cytoplasmic mtDNA was reduced both at basal levels and after H_2_O_2_ stimulation in the voltage-dependent anion channel 1/3(VDAC1/3) knockout mouse fibroblasts (MEF) when compared with wild-type MEF. Additionally, in the presence of mtDNA, VDAC1 trimer and higher-order oligomer formation was increased, suggesting that oxidatively stressed mitochondria release mtDNA fragments through pores formed in the outer mitochondrial membrane by VDAC oligomers [60]. Riley et al. treated osteosarcoma cell U2OS with ABT-737 (a BCL-xL, BCL-2 and BCL-w inhibitor), actinomycin D (ActD; an unstable transcription inhibitor) and qVD-OPh (a cysteine protease inhibitor) to promote mitochondrial apoptosis and mimic mitochondrial outer membrane permeabilization (MOMP). Moreover, mtDNA was observed by using a super-resolution Airyscan confocal microscopy in wild-type and BAX/BAK-deficient U2OS cells. They found that, under cysteine protease inhibitory conditions, which induced BAX/BAK-dependent MOMP, matrix-localized mtDNA was released from mitochondria [61]. Huang et al. transfected LPS into wild-type, *Casp11^−/−^* and *GSDMD^−/−^* mouse microvascular endothelial cells (MVEC) and found that intracellular LPS-induced mtDNA release was dependent on caspase-11 and Gasdermin D [62]. Similarly, Bao et al. detected mtDNA in the cytoplasm and mitochondrial fission, which is a sign of cellular stress, in hepatocellular carcinoma (HCC) cells treated with Drp1 [63]. Taken together, mtDNA can be released into the cytoplasm through pores formed by VDAC oligomerization, BAX/BAK-mediated MOMP and other means. In addition, Nakahira et al. extracted wild-type and *NLRP3^−/−^* mouse bone-marrow-derived macrophages, induced them with LPS, treated them with ATP and found that NALP3 also mediates mtDNA release [64]. It has been suggested that mtDNA can also be released by inflammatory vesicles. Oxidative stress in mitochondria leads to the accumulation of oxidized mtDNA fragments, which, along with ROS, are released in the cytosol as free molecules or engulfed into mitochondrial derived vesicles (MDVs). Guescini et al. demonstrated that, in human glioblastoma cells (U87MG) and astrocyte cells, exosomes can work as vesicular carriers of mtDNA [65]. Moreover, exosomes containing the complete mitochondrial genome were also detected by Sansone et al. [66]. Furthermore, Torralba et al. suggested that exosomes containing mtDNA can activate the cGAS/STING pathway in immune cells, which in turn elicits a downstream inflammatory response [67]. Accumulation of mtDNA in the cytoplasm will also spread to the extracellular space and act on nearby microglia and astrocytes (Figure 1) [68].

### 2.2. Effect of mtDNA on Microglia

The mtDNA released by neurons also has an effect on adjacent microglia. Liao et al. used mtDNA extracted from purified mitochondria, which were then transfected into cGAS knockout mice and tamoxifen-induced cGAS or HDAC3 knockout microglia, and demonstrated that the cGAS/STING pathway in microglia was activated by mtDNA and that HDAC3-regulated p65 acetylation and nuclear localization in microglia transcriptionally activated cGAS expression and enhanced cGAS/STING pathway activation, which exacerbated the inflammatory microenvironment [69]. Mathur et al. found that *STING^gt/gt^* was not sensitive to ganciclovir (GCV)-induced interferon responses compared with microglia from wild-type mice [70]. Tsilioni extracted serum isolates from 20 clinical patients with brain-inflammation-induced extracellular vesicles, and the inflammatory group had more mtDNA in cultured microglia and secreted more of the pro-inflammatory cytokine interleukin IL-1β when compared with the normal group [71]. The accumulation of oxidative damage to linear mtDNA leads to the increased production of ROS, which activates redox-sensitive NF-κB to cause excessive neuroinflammation [72]. Thus, mtDNA can enhance microglia polarization toward M1 and exacerbate the inflammatory response. In contrast, Nasi et al. treated human microglia HMC3 cells with mtDNA extracted from Hela cells and found decreased mRNA expression of IL-1β [73]. However, the majority of studies have suggested that mtDNA promotes the secretion of pro-inflammatory factors by microglia, which thereby exacerbates neuroinflammation and thus furthers the development of neurodegenerative diseases.

### 2.3. Effect of mtDNA on Astrocytes

Mitochondrial oxidative stress is an important factor leading to the disruption of mtDNA integrity and mtDNA release, which in turn may act on surrounding astrocytes by entering the cytoplasm or being secreted extracellularly via vesicles/exosomes, permeability pores and other means. In a variety of neurodegenerative diseases, TAR DNA/RNA binding protein 43 (TDP-43) exhibits aberrant aggregation and localization within neurons and glial cells. It has been shown that TDP-43 can cause dysregulation of mitochondrial proteins, leading to mitochondrial oxidative stress [74]. Yu et al. used TDP-43 transgenic mice to demonstrate that TDP-43 can invade mitochondria and release mtDNA through permeability transition pores [75]; TDP-43 has also been shown to induce astrocyte inflammation [76]. Hu found that hypoxia-induced mtDNA damage could lead to apoptosis in mouse astrocytes [77]. Ignatenko et al. used replicative mtDNA decapping enzyme Twinkle (TwKO) inactivation to induce mtDNA depletion in mouse neurons and astrocytes and found that mtDNA deletion caused astrocyte overactivation to induce early onset neurological disease [78]. The above studies suggest that the mild stress caused by mtDNA causes astrocytes to secrete more inflammatory factors, thus promoting neuroinflammation, while exacerbating the development of neurodegenerative diseases. In contrast, when mtDNA is excessively damaged or even absent, it may cause astrocytes to undergo apoptosis, which would slow down inflammation and possibly delay the onset of neurodegenerative disease.

### 2.4. mtDNA Promotes Activation of the cGAS/STING Pathway

In neurodegenerative diseases, mtDNA serves as the initiation point of neuroinflammation and how it activates the inflammatory response. The mtDNA released by neuronal cells acts on nearby microglia and astrocytes via exocytosis. The cGAS/STING pathway plays a unique and critical role in neuroinflammation and neurodegenerative diseases as one of the important aberrant cytoplasmic DNA monitors. As the primary cytoplasmic DNA receptor for microglia and astrocytes, cGAS can be activated by mtDNA that is aberrantly localized in the cytoplasm. As shown in Figure 1, when mtDNA is released into the cytoplasm by stressed/damaged mitochondria, mtDNA binds to cGAS to form a complex that induces a conformational change in the active site of cGAS, which catalyzes the synthesis of cyclic GMP–AMP (cGAMP) from ATP and GTP. The cGAMP synthesized by cGAS contains two phosphodiester bonds, one between the 2′-OH of GMP and the 5′-phosphate of AMP and the other between the 3′-OH of AMP and the 5′-phosphate of GMP, and is referred to as 2′3′-cGAMP. Overall, 2′3′-cGAMP acts as a second messenger that binds and activates STING proteins on the endoplasmic reticulum membrane, inducing oligomerization to form tetramers and translocation from the endoplasmic reticulum to the endoplasmic reticulum–Golgi intermediate region. Upon cGAMP binding, STING undergoes a conformational change. The two wings of the protein are juxtaposed with the ligand located deep in the binding pocket, and the lid consisting of four antiparallel β-sheet chains is rearranged at the top of the binding pocket, producing a closed conformation. This conformational change leads to a 180° rotation of the ligand-binding region, resulting in the formation of STING oligomers by side-by-side stacking of dimeric STING molecules. After STING is activated, STING is transferred from the endoplasmic reticulum to the Golgi apparatus, where tetramerization of STING can serve as a signaling platform to recruit and activate TANK-binding kinase (TBK1), which in turn can phosphorylate the carboxy-terminal structural domain of STING, which subsequently recruits and phosphorylates interferon regulatory factor 3 (IRF3). Phosphorylated IRF3 forms a dimer that translocates to the nucleus and induces transcriptional expression of downstream type I interferon (IFN-I) and interferon-stimulated genes (ISGs), initiating the innate immune response [79]. Riley et al. treated wild-type and STING-deficient mouse endothelial SVEC cells with ABT-737 and the MCL-1 inhibitor S63845 to induce rapid mitochondrial apoptosis and found that MOMP-induced mtDNA release initiates a cGAS/STING-dependent type I interferon response [61]. Zhang et al. administered mtDNA extracted from liver tissue to wild-type and STING-deficient mice intraperitoneally and found that mtDNA activated IFN-β in a cGAS/STING pathway-dependent manner [80]. In addition, STING recruits IκB kinase (IKK), which phosphorylates the NF-κB inhibitor, IκBα, and phosphorylation of IκBα leads to NF-κB translocation to the nucleus, activating the classical NF-κB signaling pathway and inducing the expression of genes, such as tumor necrosis factor α (TNF-α) and interleukin 6 (IL-6) [79].

### 2.5. mtDNA/cGAS/STING Pathway Exacerbates Neurodegenerative Disease

The release of mtDNA due to mitochondrial dysfunction may be an important marker in neurodegenerative diseases. Leurs et al. compared the levels of free mtDNA in the cerebrospinal fluid of patients with relapsing-remitting multiple sclerosis (RRMS), patients with progressive multiple sclerosis (PMS), controls with various neurological diseases, and healthy controls and found that PMS patients had mtDNA concentrations that were nonspecifically elevated [81]. Podlesniy et al. [82] and Cervera-Carles et al. [83] also examined levels of free mtDNA in cerebrospinal fluid from clinical patients with AD versus asymptomatic patients at risk for AD and found that preclinical free mtDNA was significantly increased. Sliter et al. found that mtDNA-induced mitochondrial stress in the absence of Parkin or PINK1 led to a STING-mediated type I interferon response in mice by *STING*^gt/gt^, *Parkin*^−/−^ and *PINK1*^−/−^ mice and their hybrids, demonstrating that Parkin and PINK1 prevent the development of a cytosolic response by clearing both damaged mitochondria to prevent increases in cytosol and circulating mtDNA to prevent inflammation and neurodegeneration, thereby alleviating symptoms of PD through mitochondrial autophagy [84]. Yu et al. used transgenic mice with the *TDP-43* mutant allele *A315T*, STING-deficient mice, and the resulting progeny from crossing these two lines to demonstrate that TDP 43 releases mtDNA through the mPTP permeability pore and activates the cGAS/STING pathway thereby exacerbating ALS [85]. Jauhari et al. found in AANAT-KO mice that mtDNA release into the cytoplasm and its mediated inflammation are dependent on the cGAS/STING/IRF3 pathway [86]. mtDNA release due to mitochondrial Lon induced Cheng et al. also found that ROS induction by Lon-induced mtDNA release activates the STING/IFN pathway to induce an inflammatory response [59].

In neurodegenerative diseases, neuronal oxidative stress randomly induces stress and damage to internal mitochondria, which respond to this stress, thereby causing an increase in neuronal response to metabolism and an increase in ROS production by the respiratory chain complex that disrupts the integrity of mtDNA to be released from the internal mitochondria. A portion of the damaged mtDNA that accumulates in the cytoplasm activates the intracellular cGAS/STING pathway to promote the secretion of pro-inflammatory factors such as IL-1β by neurons to promote the inflammatory phenotype of microglia and astrocytes near neurons, as well as to recruit leukocyte infiltration in response to neuroinflammation. In addition, most of the mtDNA may be released extracellularly via exosome vesicles and acts on neighboring microglia and astrocytes, which take up the mtDNA via endocytosis or binding to the corresponding receptors. This uptake activates the cGAS/STING pathway within these immune cells and, thus, type-I-interferon-mediated immune responses, as well as the classical NF-κB signaling pathway, to promote secretion of pro-inflammatory factors TNF-α, IL-6. The action of mtDNA released by neurons on themselves and surrounding microglia and astrocytes maintains the microenvironment of neuroinflammation.

## 3. The IL-1 Family Plays an Important Role in the Formation of the Inflammatory Environment in Neurodegenerative Diseases

All members of the IL-1 family are expressed by endogenous brain cells but at low levels. High levels of IL-1β and IL-18 are found in a variety of neurodegenerative diseases [87]. When the brain receives an inflammatory stimulus, the first cells to express IL-1 are microglia, but other brain cells (astrocytes, invading immune cells, vascular cells, and possibly neurons) can also produce IL-1. When acting on healthy neurons in vitro or in vivo, IL-1 has no toxic effects. Regulation of the mtDNA/cGAS/STING pathway by the IL-1 family is generally considered to be an inflammatory amplification signal that promotes activation of its various components, and in an inflammatory environment where neurons are present with immune cells, the IL-1 family acts as a feedback mediator from immune cells to neurons exacerbating their damage. Aarreberg et al. found that IL-1β-treated cells demonstrated a significant 3-to-6-fold enrichment of mtDNA in the cytosolic fraction compared to mock-treated cells and activation of the cGAS/STING pathway phosphorylated IRF3 at the essential activation residue serine 386 (S386) promoting inflammation [88]. It has been shown that the IL-33-induced inflammatory response is dependent on aberrantly localized double-stranded DNA (dsDNA) and regulates the subsequent inflammatory response via cGAS [89]. IL-33 levels were also increased after mtDNA treatment with cGAMP [90,91]. The above studies suggest that the IL-1 family plays a role in the mtDNA/cGAS/STING pathway to amplify signals that generate more inflammation and cause more severe damage in neurodegenerative diseases that are already in a neuroinflammatory environment.

## 4. Conclusions

**Figure 2:**
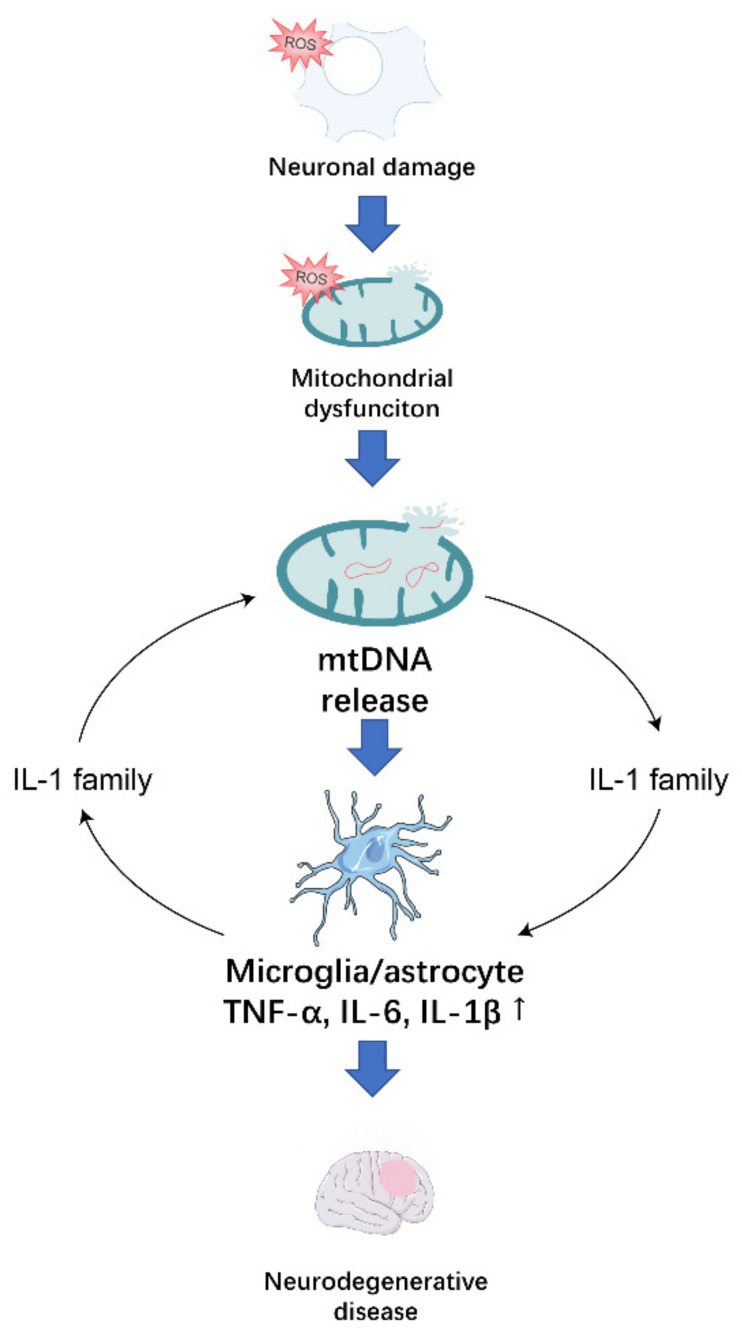
Oxidative stress in neurons induces oxidative stress in their mitochondria, leading to the release of the mtDNA, which stimulates the release of pro-inflammatory factors from surrounding microglia and astrocytes. The IL-1 family amplifies inflammatory signals in the neuroinflammatory microenvironment in which they are located, contributing to the development of neurodegenerative disease.

In conclusion, inflammation creates an essential microenvironment for the development of neurodegenerative diseases. In neurodegenerative diseases, neurons receive oxidative stress that causes mitochondrial dysfunction, and the damaged mitochondria promote the release of mtDNA, which acts on surrounding microglia and astrocytes, causing an immune response (Figure 2). Therefore, it is imperative at the inflammation microenvironment to investigate means of treating neurodegenerative diseases that target the release of mtDNA caused by mitochondrial dysfunction.

Currently, there is a relative lack of research on how the IL-1 family specifically affects the mtDNA/cGAS/STING pathway. Our previous studies have shown that IL-33 can regulate macrophage metabolism by influencing its polarization [92,93], and therefore the role of IL-33 on mtDNA and microglia in neurodegenerative diseases is worthy of our next investigation. In the neuroinflammatory microenvironment, microglia and macrophages are not only present in the M1/M2 phenotype, but they also secrete pro- and anti-inflammatory factors that balance the microenvironment. It is also noteworthy that the inflammatory environment is the result of the interaction of multiple types of cytokines, not only of the IL-1 family. Therefore, the exploration of the neuroinflammatory microenvironment remains a challenge. Furthermore, cGAS contains DNA structural domains that can recognize negatively charged dsDNA; therefore, this electrostatic interaction-dependent binding makes the recognition of DNA by cGAS non-sequence specific. It is suggested that therapeutic approaches that block the recognition of mtDNA by cGAS should also consider how to prevent DNA-induced damage from other pathogens [94]. In addition, there may be some limitations to the activation downstream of STING. For example, mutant p53 can bind to TBK1 and prevent the formation of a trimeric complex between TBK1, STING and IRF3, blocking the nuclear translocation and transcriptional activity of IRF3 [95].

Currently, inhibition of harmful proteins and aggregation is the mainstay of treatment for neurodegenerative diseases. However, whether there is a causal relationship between the phenomenon of abnormal protein or peptide aggregation and the occurrence of neuronal loss and synaptic damage is still controversial. Recently, therapeutic strategies to improve the brain’s energy metabolism to combat neurodegenerative diseases have also been an emerging therapeutic concept, and therefore mitochondria play an irreplaceable role as a source of brain function. In this review, we proposed that mitochondrial ROS-induced mitochondrial dysfunction, resulting in the release of mtDNA, may be a key mechanism contributing to neurodegenerative diseases. However, due to the complexity of mitochondrial ROS metabolism, good efficacy may not be obtained with only one antioxidant. Therefore, a more complete approach is needed in the future tools of antioxidant therapy to reduce the damage to neurons caused by ROS. Moreover, mtDNA is a central point of regulation of neuroinflammation in the microenvironment of neuroinflammation, where microglia interact strictly with neurons and their differential activation. Disruption of the homeostasis of the inflammatory microenvironment may lead to altered phenotypes of microglia. Targeting the mtDNA/cGAS/STING pathway to reverse the phenotypic changes of microglia and astrocytes may be a new strategy for the treatment of neurodegenerative diseases.

## Figures and Tables

**Table 1:** Advances in research on antioxidants in neurodegenerative diseases.

Antioxidants	Research Progress	Antioxidant Mechanism	Relative Pathway	Diseases	References
Selegiline/Rasagiline	Clinical applications	Monoamine oxidase type B (MAO-B) inhibitor		PD	[13]
GBE	In Vivo and in vitro models; clinical trials	Free radical–scavenging action	JNK, ERK1/2, Akt	AD	[14,15]
Anthocyanins extracted	In Vivo models	Free radical–scavenging action	PI3K/Akt/Nrf2	AD	[16,17]
Resveratrol	In Vivo and in vitro models	Maintaining the levels of antioxidant enzymes; free radical–scavenging action	AMPK, PI3K/Akt/GSK-3β	AD, PD, ALS	[18,19]
Coenzyme Q10	In Vivo and in vitro models; clinical trials	Antioxidant in mitochondria and lipid membranes		PD, ALS	[20,21,22]
Rutin	In vitro and in vivo models	Directly scavenge ROS	JNK, p38 MAPK,	AD, PD	[23]
SFN	In vitro and in vivo models	Free radical–scavenging action	Nrf2/ARE	AD, PD, ALS	[24,25]
Flavonoids	In Vivo models	Free radical–scavenging action	NF-κB/iNOS	AD, PD, ALS	[26,27,28]
H_2_S	In vitro and in vivo models	Mediating the activities of glutathione peroxidase, SOD and catalase	Akt/Nrf2/GSK-3β,NO pathway	AD, PD, ALS	[29,30,31,32,33]
tBHQ	In Vivo models	An Nrf2 Stabilizer	NF-κB/HSP70, PI3K/Akt	AD, ALS	[34,35,36,37]
DMF	In vitro and in vivo models	An Nrf2 activator	p62/Keap1/Nrf2/ARE, NF-κB	AD, PD, ALS	[38,39,40,41]
EGCG	In vitro and in vivo models	Free radical–scavenging action	ATF4/PTP1B, TLR4/NF-κB	AD, ALS	[42,43,44,45,46]

Abbreviations: GBE, *Ginkgo biloba* extract; JNK, c-Jun N-terminal kinase; ERK1/2, extracellular regulated protein kinases 1/2; Akt, protein kinase B; PI3K, phosphoinositide 3 kinase; AMPK, adenosine 5‘-monophosphate-activated protein kinase; GSK-3β, glycogen synthase kinase-3β; p38 MAPK, mitogen-activated protein kinases; SFN, sulforaphane; Nrf2, nuclear-factor-erythroid-2-related factor 2; ARE, antioxidant response element; NF-κB, redox-sensitive nuclear factor-κB; iNOS, inducible nitric oxide synthase; H2S, hydrogen sulfide; NO, nitric oxide; tBHQ, tert-Butylhydroquinone; HSP70, heat shock protein 70; DMF, dimethyl fumarate; Keap1, Kelch-like ECH-associating protein 1; EGCG, epigallocatechin gallate; ATF4, activation factor 4; PTP1B, protein tyrosine phosphatase 1B; TLR4, Toll-like receptor 4.
